# *Tiliacora triandra* (Colebr.) Diels Leaf Aqueous Extract Inhibits Hepatic Glucose Production in HepG2 Cells and Type 2 Diabetic Rats

**DOI:** 10.3390/molecules26051239

**Published:** 2021-02-25

**Authors:** Tipthida Pasachan, Acharaporn Duangjai, Atcharaporn Ontawong, Doungporn Amornlerdpison, Metee Jinakote, Manussabhorn Phatsara, Sunhapas Soodvilai, Chutima Srimaroeng

**Affiliations:** 1 Department of Physiology, Faculty of Medicine, Chiang Mai University, Chiang Mai 50200, Thailand; tp.pasachan@gmail.com; 2 Division of Physiology, School of Medical Sciences, University of Phayao, Phayao 56000, Thailand; achara.phso@gmail.com (A.D.); atcharaporn.on@up.ac.th (A.O.); 3 Centre of Excellence in Agricultural Innovation for Graduate Entrepreneur and Faculty of Fisheries Technology and Aquatic Resources, Maejo University, Chiang Mai 50290, Thailand; doungpornfishtech@gmail.com; 4 School of Human Kinetics and Health, Faculty of Health Science Technology, HRH Princess Chulabhorn College of Medical Science, Chulabhorn Royal Academy, Bangkok 10210, Thailand; metee.jin@cra.ac.th; 5 Department of Anatomy, Faculty of Medicine, Chiang Mai University, Chiang Mai 50200, Thailand; msethadavit@gmail.com; 6 Research Centre of Transport Protein for Medical Innovation, Department of Physiology, Faculty of Science, Mahidol University, Bangkok 10400, Thailand; sunhapas.soo@mahidol.ac.th; 7 Excellent Centre for Drug Discovery, Mahidol University, Bangkok 10400, Thailand

**Keywords:** *Tiliacora triandra* (Colebr.) Diels, diabetes, hepatic insulin resistance, hepatic gluconeogenesis, antioxidant

## Abstract

This study investigated the effects of *Tiliacora triandra* (Colebr.) Diels aqueous extract (TTE) on hepatic glucose production in hepatocellular carcinoma (HepG2) cells and type 2 diabetic (T2DM) conditions. HepG2 cells were pretreated with TTE and its major constituents found in TTE, epicatechin (EC) and quercetin (QC). The hepatic glucose production was determined. The in vitro data were confirmed in T2DM rats, which were supplemented daily with 1000 mg/kg body weight (BW) TTE, 30 mg/kg BW metformin or TTE combined with metformin for 12 weeks. Results demonstrate that TTE induced copper-zinc superoxide dismutase, glutathione peroxidase and catalase genes, similarly to EC and QC. TTE decreased hepatic glucose production by downregulating phosphoenolpyruvate carboxykinase (PEPCK) and glucose-6-phosphatase (G6Pase) and increasing protein kinase B and AMP-activated protein kinase phosphorylation in HepG2 cells. These results correlated with the antihyperglycemic, antitriglyceridemic, anti-insulin resistance, and antioxidant activities of TTE in T2DM rats, similar to the metformin and combination treatments. Consistently, impairment of hepatic gluconeogenesis in T2DM rats was restored after single and combined treatments by reducing PEPCK and G6Pase genes. Collectively, TTE could potentially be developed as a nutraceutical product to prevent glucose overproduction in patients with obesity, insulin resistance, and diabetes who are being treated with antidiabetic drugs.

## 1. Introduction

Type 2 diabetes mellitus (T2DM) refers to a group of metabolic diseases defined by hyperglycemia and insulin resistance [1]. During hyperglycemia, pancreatic β-cells over-secrete insulin, but it is not able to regulate glucose metabolism due to the presence of insulin resistance; this results in elevated blood glucose levels in T2DM [2]. Hence, besides hyperglycemia, T2DM is also characterized by excessive hepatic glucose production [3]. At present, investigating the mechanisms responsible for excess hepatic gluconeogenesis, how it drives fasting hyperglycemia in diabetes, and potential preventative and therapeutic strategies have received substantial attention [4].

In type 2 diabetes, hepatic gluconeogenesis is higher than normal in the post-absorptive state and fails to be properly suppressed by insulin, resulting in excessive glucose production rather than glycogenolysis [5]. The key enzymes involved in the regulation of gluconeogenesis are known, including phosphoenolpyruvate carboxykinase (PEPCK) and glucose-6-phosphatase (G6Pase) [6]. The expression of these enzymes is highly regulated by glucagon and insulin in correlation with maintaining blood glucose levels [7]. Expression of PEPCK has been shown to be dysregulated with a seven-fold upregulation in diabetic mice [8]. Moreover, it has been revealed that the mRNA expression of PEPCK and G6Pase are increased by the activation of peroxisome proliferator-activated receptor coactivator 1-alpha (PGC-1α) in diabetic KK-Ay mice [9]. PEPCK and G6Pase, therefore, are thought to be the rate-limiting enzymes for gluconeogenesis and have been implicated as potential targets to reduce hepatic glucose production and blood glucose levels in T2DM. Previous studies have shown that the inhibition of PEPCK by 3-mercaptopicolinic acid results in hypoglycemia in rats [10]. In addition, phosphorylation of protein kinase B (Akt) by binding at Ser473 activates the transcription factor forkhead box protein O1 (FOXO1), which, in turn, inhibits the expression of both PEPCK and G6Pase in high-fat diet (HFD)-fed mice and in hepatocellular carcinoma (HepG2) cells [11]. Activation of AMP-activated protein kinase (AMPK) by metformin, the first-line drug for T2DM, is able to suppress hepatic gluconeogenesis in T2DM rats [12].

*Tiliacora triandra* (Colebr) Diels (TT), or Yanang in Thai, a species of *Tiliacora* genus in the family Menispermaceae and class Magnoliopsida, is a native plant of Southeast Asia and widely used in northeastern Thai cuisine [13]. A previous study has identified TT methanolic extract, which is composed of hydrocarbon compounds, including vitamin E, phytol, 1-cyclohexenylacetic acid, oleamide, and oleic acid, respectively, using gas chromatography-mass spectrometry analysis [14]). On the other hand, the fingerprint of *Tiliacora triandra* leaf water or aqueous extract (TTE) revealed a high content of phenolic compounds, including gallic acid (GA), cyanidin (CD), and quercetin (QC) [15], respectively, while catechin was also found [16] using gradient high-performance liquid chromatography (HPLC). Several studies have shown that methanol or ethanol extracted from TT leaves has various pharmacological effects, such as antioxidant, hypoglycemic and inhibition of cholesterol absorption activities, while TTE showed higher antioxidant and neuroprotective effects [13,14,15,16,17,18]. Nonetheless, the hepatoprotective effect of *Tiliacora triandra* leaf aqueous extract (TTE) on hepatic glucose production in the T2DM model has not been investigated yet. Thus, this study assessed the antidiabetic effects of TTE on hepatic gluconeogenesis and identified the mechanism involved in both HepG2 cells and T2DM rat models. These findings could prove insightful for developing TTE into potential nutraceutical or pharmaceutical products for the treatment of type 2 diabetes and prevention of its complications, including non-alcoholic fatty liver disease, non-alcoholic steatohepatitis, and cirrhosis.

## 2. Results

### 2.1. Quantification of Major Constituents Present in TTE and Their Phenolic Contents and the Radical Scavenging Activities

**Figure 1:**
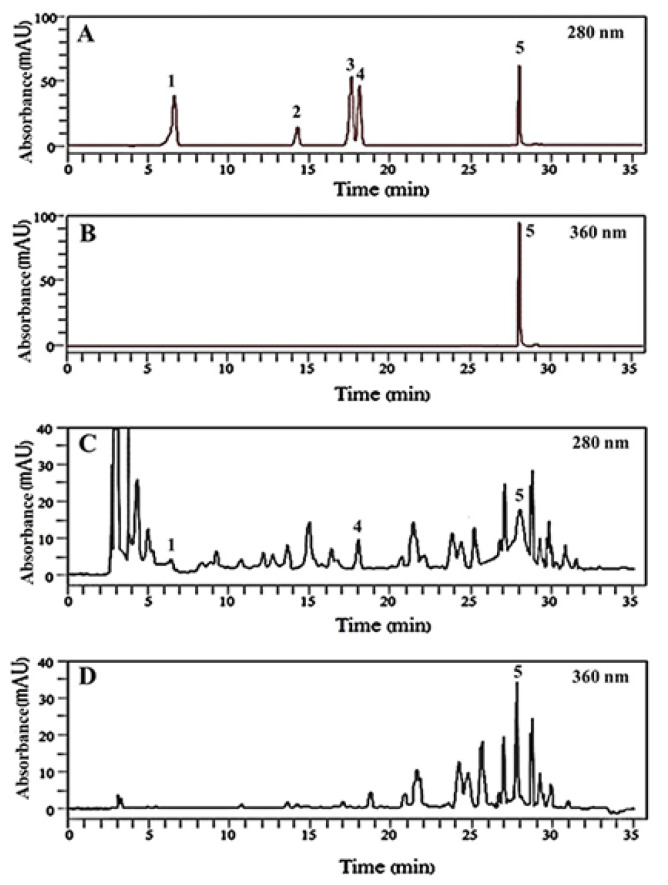
Chromatograms obtained in the HPLC-photo diode array (PDA) analysis of Diels aqueous extract (TTE). HPLC chromatograms of standard solutions at (**A**) 280 nm, (**B**) 360 nm, (**C**) TTE sample at 280 nm and (**D**) TTE sample at 360 nm using the solvent gradient program, as shown in Supplementary Table S2. Peaks: 1, gallic acid. (6.45 min); 2, catechin (14.52 min); 3, (–)-epigallocatechin gallate (17.63 min); 4, epicatechin (18.35 min); 5, quercetin (28.15 min).

As shown in Table 1, the major polyphenolic compounds in TTE were analyzed using HPLC-PDA compared with the calibration curve (Figure 1A–C and Supplementary Table S1). The HPLC chromatograms of TTE reliably detected gallic acid, EC, and QC (Figure 1 and Table 1). A high level of EC was detected at 1.37 ± 0.025 mg/g extract, whereas QC and gallic acid were found at minimal levels of 0.134 ± 0.001 and 0.053 ± 0.007 mg/g extract, respectively. Catechin and (−)-epigallocatechin gallate (EGCG) were undetectable (Table 1).

The total phenolic content of TTE was 30.36 ± 0.16 mg gallic acid equivalents (GAE)/g extract, which was less than that found in active compounds like EC and QC (Table 2). Correspondingly, the EC_50_ value of TTE against 2,2′-azino-bis-3-ethylbenzthiazoline-6-sulfonic acid (ABTS) radical was 324.20 ± 1.62 µg/mL. This result agreed with the EC_50_ value obtained by the diphenyl-1-picrylhydrazyl (DPPH) radical scavenging activity at 20.30 ± 0.05 µg/mL. Furthermore, EC and QC exhibited comparable scavenging activities when compared with that of TTE, as shown in Table 2, which suggests that TTE has a strong antioxidant capacity as it contains polyphenols, including EC and QC.

### 2.2. Effect of TTE on Oxidative Stress in HepG2 Cells

**Figure 2:**
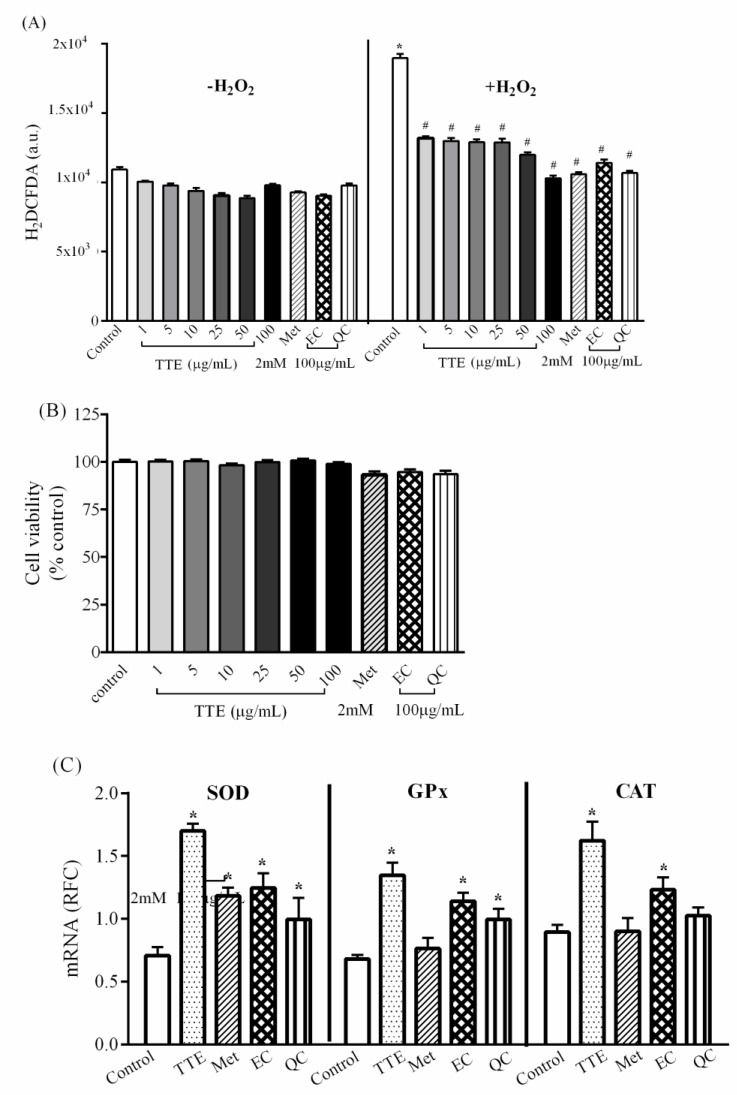
The effect of TTE on H_2_O_2_-induced oxidative stress and cell viability in HepG2 cells. (**A**) HepG2 cells were pretreated with 100 µg/mL TTE, epicatechin (EC), quercetin (QC), and 2 mM metformin (Met) for 24 h. The cells were subsequently incubated with H2DCFDA. The fluorescence DCF was subsequently detected. * *p* < 0.05 vs. control without H_2_O_2_, # *p* < 0.05 vs. cells treated with H_2_O_2_ alone. (**B**) The cells were incubated with 1–100 µg/mL TTE, EC, QC, and 2 mM metformin (Met) for 24 h, and the cell viability was assessed using MTT assay. (**C**) mRNA expression levels of antioxidant genes, including copper-zinc superoxide dismutase (Cu-ZnSOD), glutathione peroxidase (GPx), and catalase (CAT) in HepG2 cells. Total RNA was extracted from HepG2 cells, and mRNA levels were measured using quantitative real-time PCR (qPCR) The results are reported as relative fold change (RFC) and expressed as mean ± standard error of mean (SEM) (*n* = 5). * *p* < 0.05 vs. control cells.

To identify the antioxidant effect of TTE and its major constituents, EC and QC, which exert an antihyperglycemic effect and improve insulin resistance, total reactive oxygen species (ROS) in HepG2 cells were determined using 20,70-dichlorfluoresceindiacetate (H_2_DCFDA) dye. As shown in Figure 2A, 100 µg/mL of TTE, EC, and QC did not change ROS levels under normal conditions, similar to 2 mM metformin, indicating that there was no effect of TTE on the generation of intracellular free radicals. H_2_O_2_ significantly increased ROS production and oxidative stress when compared with control, while pre-incubated with TTE at 1–100 µg/mL or 100 µg/mL EC and QC, significantly inhibited ROS production similar to the antidiabetic drug metformin. In addition, this antioxidant effect of TTE, its major components, and metformin did not affect the viability of HepG2 cells, as shown by the 3-(4,5-dimethylthiazol-2-yl)-2,5-diphenyltetrazolium bromide (MTT) assay (Figure 2B), suggesting that TTE and its major constituents have antioxidant effects by scavenging ROS. Besides this ROS scavenging property, TTE also demonstrated antioxidant properties by upregulating genes related to antioxidant enzymes, i.e., copper-zinc superoxide dismutase (Cu-ZnSOD), glutathione peroxidase (GPx), and catalase (CAT), similar to its major constituent EC, as shown in Figure 2C. In addition, 100 µg/mL QC also upregulated Cu-ZnSOD and GPx gene expression, while 2 mM metformin only increased SOD transcripts. Thus, these data indicate that TTE, partly via EC and QC, has antioxidant capacity by scavenging free radicals and modulating antioxidant gene expression.

### 2.3. Effect of TTE on Glucose Production in HepG2 Cells

**Figure 3:**
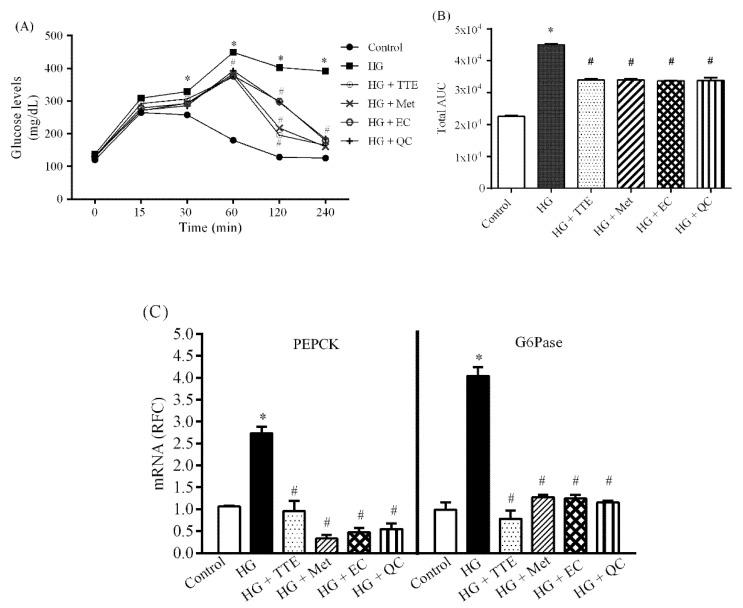
The inhibitory effect of TTE on hepatic glucose production in HepG2 cells. Cells were incubated with or without a medium containing 5 mM glucose (HG) for 24 h. Subsequently, HG cells were treated with 20 mM sodium lactate and 2 mM sodium pyruvate for another 240 min. (**A**) The glucose levels in the media in each condition, including HG cells treated with 100 µg/mL TTE (HG + TTE), HG cells treated with 2 mM metformin (HG + Met), HG cells treated with 100 µg/mL epicatechin (HG + EC), and HG cells treated with 100 µg/mL quercetin (HG + QC) were determined using a commercial colorimetric assay. (**B**) Total area under the curve was calculated from (**A**). (**C**) Phosphoenolpyruvate carboxykinase (PEPCK) and G6Pase mRNA expression from each condition were measured using quantitative real-time PCR (qPCR). The results are reported as RFC and expressed as mean ± SEM (*n* = 5). * *p* < 0.05 vs. the control group, # *p* < 0.05 vs. the HG group.

To further investigate whether the antioxidant capacity of TTE exerts hepatoprotective effects by suppressing hepatic glucose production, hepatic gluconeogenesis induced by a mixture of amino acids was examined in HepG2 cells. As shown in Figure 3A, high glucose (HG)-treated cells significantly increased glucose production after challenge with the lactate/pyruvate mixture for up to 240 min when compared with control, suggesting that hyperglycemia and insulin resistance impair hepatic gluconeogenesis. Nonetheless, TTE, metformin, EC, and QC improved pyruvate tolerance, as represented by the significant reduction in the total area under the curve (AUC) (Figure 3B).

To identify the mechanism by, which TTE modulates hepatic gluconeogenesis, the expression of PEPCK and G6Pase (rate-limiting enzymes for gluconeogenesis) was also determined. As expected, HG cells upregulated PEPCK and G6Pase mRNA expression compared with control (Figure 3C), indicating that hyperglycemia enhanced hepatic gluconeogenesis. In contrast, TTE, EC, QC, and metformin reduced the expression of these two genes (Figure 3C). Thus, TTE, partly via EC and QC, as well as metformin, can suppress hepatic gluconeogenesis, leading to improved hyperglycemia under diabetic conditions.

### 2.4. Effect of TTE on Insulin Signaling in HepG2 Cells

**Figure 4:**
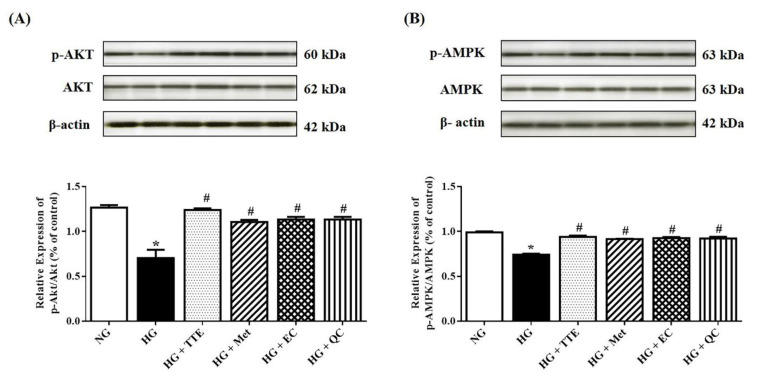
Effect of TTE on hepatic insulin signaling in HepG2 cells. (**A**) p-Akt and total Akt protein expression and (**B**) p-AMPK and total AMPK protein expression in HepG2 cells in each condition, including HG cells treated with 100 µg/mL TTE (HG + TTE), HG cells treated with 2 mM metformin (HG + Met), HG cells treated with 100 µg/mL epicatechin (HG + EC), and HG cells treated with 100 µg/mL quercetin (HG + QC). A representative blot of p-AMPK, AMPK, p-Akt, and total Akt protein expression is shown on the top panel and quantification of relative protein expression in each fraction is presented on the bottom. Anti-β-actin antibody was used as loading control. Values shown are mean ± SEM (*n* = 3), * *p* < 0.05 vs. the control group, # *p* < 0.05 vs. the HG group.

A previous study has shown that the activation of Akt/AMPK inhibits the expression of the hepatic gluconeogenic enzymes, PEPCK and G6Pase [4]. We, therefore, further investigated the effect of TTE on phosphorylation of Akt and AMPK signaling pathway using Western blot analyses. As shown in Figure 4, the phosphorylation of AMPK (Thr^172^) and Akt (Ser^473^) was significantly decreased in the HG group when compared with the control. On the other hand, treatment of HepG2 cells with TTE at 100 µg/mL increased the phosphorylation of AMPK and Akt compared with HG cells; similar effects were seen with EC, QC, and metformin treatment. These data suggest that TTE, mainly via EC and QC, is able to improve hyperglycemia, insulin resistance, and hepatic gluconeogenesis, similar to the effects of metformin.

### 2.5. Effects of TTE on General Characteristics, Glucose Tolerance, and Liver Morphology in T2DM Rats

**Figure 5:**
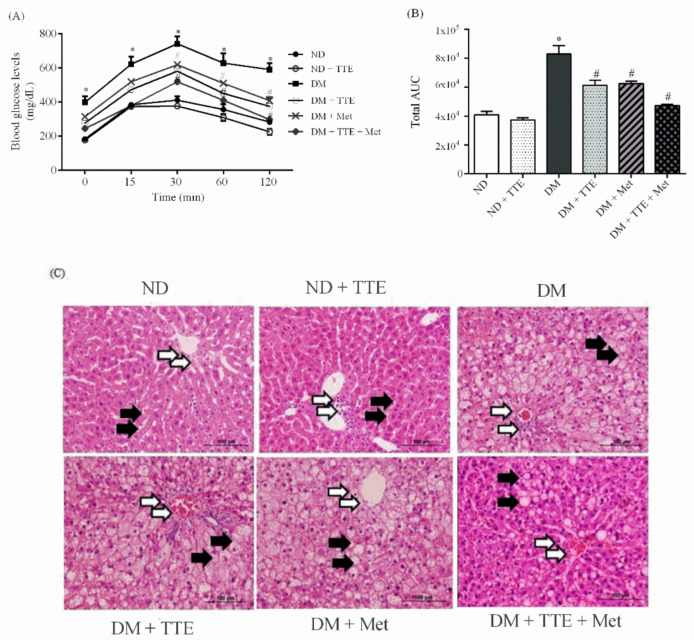
The effect of TTE on glucose intolerance in type 2 diabetic (T2DM) rats. Blood glucose levels were measured at the basal level and after glucose loading (1 g/kg BW) for 120 min. (**A**) Plasma glucose levels were determined using a commercial colorimetric assay. (**B**) Total area under the curve (AUC) from (**A**). Data are expressed as mean ± SEM (*n* = 6), * *p* < 0.05 vs. the ND group, # *p* < 0.05 vs. the T2DM group. (**C**) Micrographs of conventional hematoxylin and eosin (H&E) staining of the liver tissues. The liver from each experimental group was removed, fixed, embedded, cut, and stained by H&E (original magnification 100×). The assay was performed at least three times on separate sets of animals. The data were then analyzed using bright-field microscopy. White arrow indicates inflammation, dilation in the central vein, fibrosis, and leukocytic infiltration around the central vein. Black arrow indicates fat vacuoles in liver tissues.

As shown in Table 3, type 2 diabetes mellitus (T2DM) rats had a significant increase in body weight (BW), BW gain, liver weight (LW), and visceral fat when compared with the ND group, although the LW/BW ratio was not different among experimental groups. In contrast, these parameters were markedly lower in the DM + TTE, DM + Met, and DM + TTE + Met groups when compared with T2DM alone, suggesting that TTE was able to control BW in T2DM. Furthermore, plasma glucose and triglyceride levels were significantly higher in T2DM rats when compared with ND. Again, these parameters were significantly reduced in DM + TTE, DM + Met, and DM + TTE + Met rats when compared with the T2DM group. Although there was no significant difference in plasma insulin among the experimental groups, the HOMA index, which indicates insulin resistance, was significantly increased in T2DM rats; this parameter was decreased by TTE, metformin, and the combination treatment. Interestingly, the reductions in plasma glucose, the HOMA index, and triglycerides were most profound with DM + TTE + Met treatment compared to single treatment with TTE or metformin, implying that TTE additively improved hyperglycemia, hypertriglyceridemia, and insulin resistance in T2DM rats treated with metformin. Similar to the general characteristics of T2DM, these results show that T2DM rats exhibited glucose intolerance, represented by a significant AUC, whereas T2DM rats treated with TTE, metformin, or the combination treatment had a significantly decreased AUC when compared with T2DM rats (Figure 5A,B). These findings suggest that TTE has antidiabetic effects against hyperglycemia, hyperlipidemia, insulin resistance, and glucose intolerance.

To further investigate the hepatoprotective effects of TTE, a morphological analysis was performed using standard methods for tissue biopsy, i.e., hematoxylin and eosin (H&E) staining. As shown in Figure 5C, normal hepatic structures were demonstrated in ND and ND + TTE rat hepatocytes. Each lobule is made up of radiating plates, with strands of cells forming a network around a central vein. On the other hand, liver sections from T2DM rats revealed the hepatocellular injury, observed as the loss of the normal architecture of the liver, inflammation, dilation of the central vein, fibrosis, and leukocytic infiltration around the central vein. The nuclei of T2DM rat hepatocytes demonstrated pyknosis and binucleated cells with numerous and large fat vacuoles. In contrast, the liver sections from the DM + TTE, DM + Met, and combination groups showed hepatic lobules, a border of hepatocytes, reversible cell injury around the central vein, and a smaller number of fat vacuoles. This result indicates that TTE, metformin, and the combination treatment have the potential to reduce inflammation and hepatic steatosis in T2DM. Furthermore, the combination of TTE and metformin seemed to have an additive effect on the prevention of hepatic steatosis in T2DM rats, as shown by the similar liver histopathology compared to that of ND rat liver sections.

### 2.6. Effect of TTE on Genes Related to Antioxidant and Hepatic Gluconeogenesis in T2DM Rats

**Figure 6:**
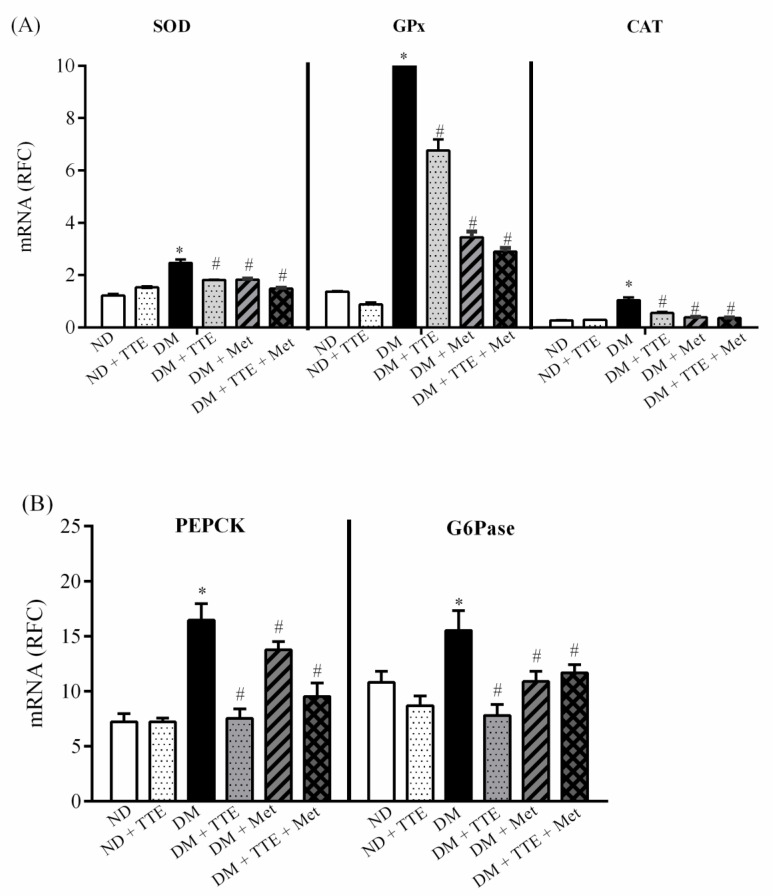
The effect of TTE on genes involved in antioxidant status and hepatic gluconeogenesis in T2DM rats. mRNA expression levels of (**A**) copper-zinc superoxide dismutase (Cu-ZnSOD), glutathione peroxidase (GPx), and catalase (CAT) and (**B**) PEPCK and G6Pase from ND, ND + TTE, DM, DM + TTE, DM + Met and DM + TTE + Met in rat liver. Results are reported as RFC and expressed as mean ± SEM (*n* = 5). * *p* < 0.05 vs. the ND group, ^#^ *p* < 0.05 vs. the DM group.

To further determine whether TTE has an antioxidant capacity and suppresses hepatic gluconeogenesis, the mRNA expression of genes involved in antioxidant and hepatic gluconeogenesis was determined using qPCR. As shown in Figure 6A, T2DM rat livers had significantly increased mRNA expression of SOD, GPx, and CAT compared with those of ND rats, indicating a compensatory response of antioxidant genes against hepatic oxidative stress in T2DM. In contrast, the expression of these three genes significantly was decreased in the DM + TTE, DM + Met, and DM + TTE + Met groups when compared with T2DM rats. These results indicate that TTE and metformin normalized oxidative stress in T2DM through the modulation of antioxidant gene transcription. This study also further investigated whether TTE regulates hepatic gluconeogenesis via the rate-limiting step enzymes, PEPCK and G6Pase. Similar to the in vitro study, hepatic PEPCK and G6Pase mRNA levels were markedly increased in the T2DM group when compared with the ND group. On the other hand, these two genes were significantly decreased in T2DM rats treated with TTE, metformin, and the combination regimen compared to levels in T2DM rats (Figure 6B), suggesting that TTE can suppress hepatic gluconeogenic activity in T2DM.

## 3. Discussion

This study demonstrates the pharmacological effect of *Tiliacora triandra* (Colebr) Diels leaf aqueous extracted (TTE) under diabetic conditions by exhibiting antidiabetic, antioxidant, and hepatoprotective effects mainly through inhibition of the gluconeogenesis pathway. Several studies have shown that the water and/or methanol and ethanol extracts of *Tiliacora triandra* (Colebr) Diels leaves have beneficial effects, such as antioxidant, neuroprotective, hypoglycemic, and inhibition of cholesterol absorption activities due to a variety of active components [13,14,15,16,17,18]. To assess the potential and safe use of TTE as a nutraceutical product to prevent hepatic glucose overproduction in diabetes, this study extracted TTE in water and identified phenolic compounds from TT leaves. It was found that TTE is rich in EC, QC, and gallic acid, whereas catechin and EGCG were undetectable. Both TTE and its major constituents exhibited strong antioxidant properties against ABTS and DPPH radicals. This study also clearly demonstrates that treatment with TTE and its major active components suppress hepatic glucose production induced by an amino acid mixture, reduced oxidative stress by scavenging free radicals, and induced antioxidative gene synthesis in hepatocytes, in which hyperglycemia induced-oxidative stress triggers the development of insulin resistance and a consequent of hepatic glucose overproduction [19]. Within this context, the higher antioxidant activity of phenolic compounds extracted from water (TTE) is also demonstrated when compared with ethanol and acetone extracts of *Tiliacora triandra* [16]. Similarly, TTE exerts antioxidant and neuroprotective effects in neurodegenerative rats induced by ethanol [15]. Although the independent study is recently shown hypoglycemic effects of TT extracted from ethanol in type 1 diabetes mellitus (T1DM) rats, the active ingredients that exhibit glucose-lowering activity have not been identified [17]. Indeed, QC, a component found in TTE, has been suggested as a powerful antioxidant by enhancing SOD and GPx activities and is able to reduce both glucose and insulin levels in T2DM rats [20]. A QC-rich onion peel extract had antihyperglycemic effects and improved insulin sensitivity via the upregulation of insulin receptor and glucose transporter 4 mRNA expression in skeletal muscle tissues in T2DM [21]. In addition, QC exhibited antiobesity and anti-insulin resistance effects in the adipocytes of obese rats and resulted in the downregulation of peroxisome proliferator-activated receptor gamma (PPAR-γ) mRNA in liver, muscle, and adipose tissue in high-fat diet-induced insulin-resistant rats [22,23]. Additionally, EC has been identified as an important bioactive compound in *Pterocarpus marsupium*, a tree that is widely distributed in the central, western, and southern regions of India. This plant has been used as an important traditional medication for the treatment of diabetes and other pathologies, including cardiovascular and liver diseases [24]. Moreover, EC has direct antioxidant effects by scavenging ROS and indirect effects by modulating pathways that regulate ROS production and antioxidant enzymes [25]. Although a small fraction of each major ingredient was found in TTE, the crude TTE has a great potential to be further developed through nanotechnology as medicine and/or nutraceutical product, given the overall achievement of TTE on antihyperglycemia and anti-insulin resistance in an in vivo (type 2 diabetic rat model) shown in this study. Moreover, by using HPLC analysis, the additional powerful and sensitive technique is needed to identify and quantify other unknown bioactive compounds in this mixture, e.g., liquid chromatography-mass spectrometry (LC-MS), as a standardized compound, for upscaling TTE in alternative medicine industry.

At present, the hepatic enzymes involved in gluconeogenesis are the subject of intense focus as therapeutic targets for maintaining hepatic cellular energy and subsequently improving hepatic complications in T2DM. For instance, the activation of AMPK could suppress G6Pase and PEPCK activity, resulting in decreased hepatic glucose production. This also enhanced fatty acid oxidation and decreased hepatic glucose, cholesterol, and triglyceride syntheses in diabetic rats and reduced insulin resistance in HepG2 cells [26,27]. Similarly, this study demonstrates for the first time that TTE and its major constituents reduced PEPCK and G6Pase levels and activated Akt/AMPK, resulting in improved insulin resistance and hepatic gluconeogenesis in both in vitro and in vivo. This evidence is consistent with a previous study showing that epigallocatechin gallate activates AMPK and consequently inhibits hepatic gluconeogenesis via AMPK [28]. EC, a component found in TTE, has been reported to partly reverse the inhibition of Akt phosphorylation in high glucose-induced insulin resistance in HepG2 cells (Lin and Lin 2008). Similarly, the flavonoid naringenin was found to activate the Akt/AMPK signaling pathway and led to improved hepatic gluconeogenesis in HepG2 cells, while green tea was found to activate phosphoinositide 3-kinase/protein kinase B (PI3K/Akt) mRNA expression in the liver in insulin-resistant rats [29,30]. A combination of higher doses of streptozotocin (45 and 55 mg/kg BW) exhibited hyperglycemia, insulin deficiency, along with losing bodyweight, which, in turn, mimics T1DM [31]. In this study, however, a combination of a high-fat diet with a lower dose of streptozotocin at 40 mg/kg BW clearly demonstrated hyperglycemia, hypertriglyceridemia, glucose intolerance, insulin resistance, and body weight gain. These features are characterized as experimental T2DM models [31,32] and are similar to those observed in type 2 diabetic humans [1]. Again, 1000 mg/kg BW of TTE exerts antidiabetic and antioxidant effects similar to treatment with the antihyperglycemic drug metformin. In addition, combined treatment with TTE and metformin additively restores this impairment without any adverse effects in this animal model. Consistently, a previous study reported that TTE at 5000 mg/kg BW did not show any acute toxicity, while the concentration up to 1200 mg/kg BW has no subchronic toxicity in rats [33]. Thus, TTE rich in polyphenols has a great potential to prevent or delay the progression of T2DM. In this light, a nanotechnology application for developing TTE to nutraceutical/medicinal products is further required. Currently, there are several herbal medicines that possess antioxidant properties and could be beneficial for reducing oxidative stress, a key pathogenic factor of diabetes [34]. For example, our recent study has shown that *Cladophora glomerata* extract exhibits antioxidant, antidiabetic, and renoprotective effects [35]. Similarly, an *Alchemilla mollis* root extract showed hepatoprotective and hypoglycemic activity in diabetic rats [36].

Besides the beneficial effects of TTE are addressed, the mechanisms of action of TTE on hepatic gluconeogenesis were strongly identified in the present study. A recent report has suggested that vitamin C and the antioxidant properties of *Asparagus officinalis* extract have antidiabetic effects via several mechanisms, such as a decrease in hepatic oxidative stress and improved hepatic gluconeogenesis [37]. In addition, the antidiabetic effects of *Gynura procumbens* extract were also demonstrated by improving insulin sensitivity and suppressing hepatic gluconeogenesis in diabetic mice [38]. Likewise, *Vernonia amygdalina Delile* extract suppresses the expression of the key hepatic gluconeogenesis enzymes, PEPCK and G6Pase, in T2DM mice [39]. Of particular note, this study also clearly demonstrates the mechanism of action of TTE, partly via EC and QC, on improved hepatic glucose production and hepatoprotection in T2DM, particularly the effectiveness of TTE combined with metformin against hyperglycemia, hypertriglyceridemia, and hepatic insulin resistance. Hence, combination therapy with an antidiabetic drug and TTE may also be an option for effective hepatic prevention without toxic effects in diabetes.

## 4. Materials and Methods

### 4.1. Chemicals

Monoclonal mouse anti-β-actin was purchased from Abcam (Cambridge, MA, USA). AMP-activated protein kinase (AMPK), phosphorylated AMPK antibodies, complete protease inhibitor cocktail and metformin, were purchased from Merck (Darmstadt, Germany). Polyclonal protein kinase B (Akt) and phosphorylated Akt (Ser473) were purchased from Cell Signaling Technology (Danvers, MA, USA). CelLytic™ MT cell lysis buffer, Dulbecco’s modified Eagle’s medium (DMEM), streptozotocin (STZ), quercetin (QC), epicatechin (EC), and gallic acid (GA) were purchased from Sigma-Aldrich (St. Louis, MO, USA). Fetal bovine serum (FBS) was purchased from Gibco (Carlsbad, CA, USA). Insulin was obtained from Biocon (Bangkok, Thailand). All other chemicals were of high purity and were obtained from commercial sources, including VWR (VWR international, Radnor, PA, USA), AppliChem (AppliChem GmbH, Darmstadt, Germany), and Vivantis (Vivantis technologies Sdn Bhd, Selangor Darul Ehsan, Malaysia).

### 4.2. TTE Extract Preparation, Purification and Qualification

*Tiliacora triandra* (Colebr) Diels aqueous extract (TTE) was collected from a community enterprise at Bantum subdistrict, Muang district, Phayao, Thailand. A voucher specimen (number 003803) was deposited at the herbarium of the Faculty of Science, Naresuan University, Phitsanulok, Thailand. The plant identity was confirmed by a plant taxonomist of the Center of Excellence in Agricultural Innovation for Graduate Entrepreneur, Maejo University, Chiang Mai, Thailand. Briefly, TT dried leaves were extracted with boiling water at 100 °C for 1 h, and the TT solution was subsequently filtered through a fabric filter twice. The TT solution was dried using an SDE-100EURO 2 spray dryer (Euro Best Technology Co., Ltd., Pathumthani, Thailand), and the TT extract (TTE) was stored at −20 °C until further experiments. High-performance liquid chromatography (HPLC) with a photo diode-array detector (PDA) (Flexar LC, PerkinElmer, Shelton, USA) was used to measure the major constituents found in TTE at the Centre of Excellence in Agricultural Innovation for Graduate Entrepreneur, Maejo University. The HPLC system consisting of a quaternary pump with a vacuum degasser, thermostated column compartment, an autosampler, and PDA was used. HPLC separation was performed on a reverse-phase Brownlee^TM^ column (C18, 5 µm, 250 × 4.6 mm, PerkinElmer, Shelton, CT, USA), and the column temperature was maintained at 35 °C. The gradient program for the HPLC analysis was shown in the Supplementary Table S2. The flow rate was 1.0 mL/min, and the injection volume was 10 µL. Detection was carried out by PDA at 280 nm for gallic acid, catechin, (−)-epigallocatechin gallate (EGCG) and epicatechin (EC), whereas quercetin (QC) was detected at both 280 and 360 nm, respectively. Prior to HPLC analysis, all solutions were filtered through a 0.45 µm membrane filter and then degassed in an ultrasonic bath for 15 min. Quantification was performed by establishing calibration curves for each standard compound. The quantification was done in triplicates and expressed as mg/g of extracted sample. The data analysis was performed using Chromera software (PerkinElmer, Shelton, CT, USA).

### 4.3. Determination of Total Polyphenol Contents, 2,2′-Azino-bis-3-ethylbenzthiazoline-6-sulfonic acid (ABTS) and 2,2-Diphenyl-1-picrylhydrazyl (DPPH) Radical Scavenging Activities

The total phenolic content of TTE and its major components were quantified using Folin–Ciocâlteu reagent using a protocol modified from a previous study [40]. In brief, TTE was incubated with 10% Folin–Ciocâlteu and 7.5% Na_2_CO_3_ solution for 1 h at room temperature and detected at a wavelength of 765 nm using a Synergy^TM^ HT microplate reader (Biotek, VT, USA). Gallic acid was used for creating calibration curves, and total phenolic content was expressed as milligrams of gallic acid equivalents (GAE)/g of TTE.

In addition, the scavenging activity of TTE and its major components against 2,2′-azino-bis-3-ethylbenzthiazoline-6-sulfonic acid (ABTS) radical and diphenyl-1-picrylhydrazyl (DPPH) was modified and evaluated following previous studies [41,42]. Briefly, 1–100 µg/mL TTE and 10–50 µg/mL gallic acid, EC, and QC were mixed with ABTS reagent and absorbance was detected at a wavelength of 734 nm within 6 min. Trolox, a derivative of vitamin E, was employed as a positive control. Data were calculated from a concentration-response curve and represented as the effective concentration at which ABTS^+^ radical was scavenging by 50% (EC_50_). Similarly, TTE and its major components were mixed with 100 µM DPPH solution and incubated for 30 min at RT in dark conditions. The remaining amount of DPPH was determined at a wavelength of 517 nm.

### 4.4. Cell Culture

A human hepatocellular carcinoma cell line (HepG2) was purchased from American Type Culture Collection (ATCC) (Manassas, VA, USA). The cells were used at passages 2–9 and grown in DMEM containing 3.7 g/L NaHCO_3_ and 4.5 g/L d-glucose, supplemented with 10% fetal bovine serum (FBS) and 100 units/mL penicillin–streptomycin solution (Life Technologies, Grand Island, NY, USA) in a humidified incubator with 37 °C and 5% CO_2_ in the air and subcultured every 4–5 days using phosphate-buffered saline (PBS) containing 0.05% trypsin-EDTA solution (Life Technologies, Grand Island, NY, USA).

#### 4.4.1. Determination of Cell Viability

To evaluate the effect of TTE and its major constituents found in TTE, i.e., EC and QC, on cell viability in HepG2 cells, the 3-(4,5-dimethylthiazol-2-yl)-2,5-diphenyltetrazolium bromide (MTT) assay was performed. The cells were incubated with a serum-free medium in the absence or presence of either 1, 5, 10, 25, 50, 100 µg/mL TTE, 100 µg/mL EC and QC, which is the antioxidants that exerted antihyperglycemic effect and improved insulin resistance [20,21,22,23], found in TTE or 2 mM metformin (an antidiabetic drug as the positive control), as previously described [43] for 24 h. To identify the mechanisms by, which these major active components contributed to the suppression of hepatic gluconeogenesis, the maximum concentration of EC and QC used was 100 µg/mL. After incubation, the incubation medium was aspirated, and the medium containing 0.5 mg/mL of MTT was incubated in each well at 37 °C for 4 h. Subsequently, the MTT solution was aspirated, and the cells were washed once with ice-cold PBS. DMSO was subsequently added to each well for another 30 min. The absorbance of the dissolved formazan was measured at a wavelength of 570 nm using a Synergy^TM^ HT microplate reader (Biotek, VT, USA). The signal detected at a wavelength of 680 nm was used as a reference. The percentage of cell viability was calculated and compared with the control using Equation (1):

(1) $$\%\;\mathrm{Cell}\;\mathrm{viability}=\frac{\left(\mathrm{absorbance}\;\mathrm{values}-\mathrm{reference}\;\mathrm{values}\right)\times 100}{\mathrm{mean}\;\mathrm{of}\;\left(\mathrm{absorbance}\;\mathrm{values}-\mathrm{reference}\;\mathrm{values}\right)\mathrm{in}\;\mathrm{control}\;\mathrm{group}}$$

#### 4.4.2. Determination of Total Reactive Oxygen Species

To evaluate the antioxidant effects of TTE on intracellular reactive oxygen species (ROS), the fluorescent dye 20,70-dichlorfluoresceindiacetate (H_2_DCFDA) (Sigma-Aldrich, St. Louis, MO, USA) was used, as previously described [30]. HepG2 cells were seeded at 2 × 10^5^ cells/mL in 96-well plates and grown for 3 days. Cells at 80–90% confluence were pretreated with different concentrations of TTE (1–100 µg/mL), 100 µg/mL EC and QC or 2 mM metformin for 24 h. Subsequently, the cells were washed and incubated with 10 µM H_2_DCFDA for 1 h at 37 °C in the dark. H_2_O_2_ (0.5 mM) was then added to each well and incubated for another 30 min. At the end of the experiment, the cells were washed twice, and intracellular ROS was measured using a Synergy^TM^ HT microplate reader at an excitation wavelength of 495 nm and an emission wavelength of 527 nm.

#### 4.4.3. Determination of Glucose Production in HepG2 cells

To evaluate the effect of TTE and its major constituents on hepatic gluconeogenesis, HepG2 cells were cultured in 24-well plates in the DMEM containing 5 and 25 mM glucose, which represents control and high glucose (HG) conditions, respectively. HepG2 cells in both conditions were seeded at 5 × 104 cells per well in 24-well plates and grown for 2 days. Subsequently, HG cells were treated with the maximum and effective dose of 100 µg/mL TTE, EC, QC, or 2 mM metformin for another 24 h. Hepatic glucose production in HepG2 cells was then assessed by incubating with 20 mM sodium lactate and 2 mM sodium pyruvate for 2 h. The medium was collected at 15, 30, 60, 120, and 240 min in order to evaluate the rate of glucose production by measuring glucose levels using an enzymatic colorimetric assay kit obtained from Erba Lachema (Brno, Czech Republic).

### 4.5. Animals

Male Wistar rats weighing 120–150 g each were obtained from the National Laboratory Animal Centre, Mahidol University (Salaya, Thailand). The animal facilities and protocols were approved by the Laboratory Animal Care and Use Committee at the Faculty of Medicine, Chiang Mai University, Chiang Mai, Thailand (38/2557). All rats were housed in a room maintained at 25 ± 1 °C on a 12:12 h dark–light cycle. The 36 rats were randomly divided into two groups; 12 and 24 rats for normal and high-fat diet groups, respectively. Normal-diet fed rats consumed a commercially available normal chow diet (C.P. Mice feed food no. 082, Bangkok, Thailand), with fat representing 19.77% of the total energy in the diet (% E); high-fat diet rats received a high-fat diet containing approximately 59.28% energy from fat, ad libitum. After 2 weeks of dietary manipulation, the rats were induced as an experimental type 2 diabetic model using a modified method as previously described [31]. Briefly, the high-fat diet rats were intraperitoneally injected (i.p.) with a single dose of 40 mg/kg BW of STZ dissolved in 0.1 M citrate buffer (pH 4.5), while the normal diet group was given citrate buffer i.p. fourteen days later, the rats with a fasting blood glucose level exceeding 250 mg/dL were considered to have type 2 diabetes. Subsequently, the animals were equally and randomly divided into six groups: normal diet (ND), a normal diet supplemented with TTE at a dose of 1000 mg/kg BW (ND + TTE), T2DM (DM), T2DM supplemented with TTE at a dose of 1000 mg/kg BW, T2DM treated with metformin at a dose of 30 mg/kg BW (DM + Met) similar to our previous report [35], and T2DM supplemented with TTE and metformin (DM + TTE + Met). This single high dose of TTE was selected based on a preliminary study, which showed that using an oral glucose tolerance test in normal rats, TTE at 1000 mg/kg BW was able to reduce glucose levels similar to that of 30 mg/kg BW metformin, while 500 mg/kg BW TTE did not (data not shown). Moreover, a previous study demonstrated that 20 mg/kg BW of EC improves the lipid profile and insulin resistance in high-fructose-fed rats [44]. Thus, 1000 mg/kg BW of TTE, containing approximately 1.37 mg EC or metformin, was administered daily by oral gavage starting at week 13 and continued for the next 12 weeks. In order to avoid a TTE-metformin interaction, rats from the combination group were given metformin by oral gavage; TTE was administered 1 h later, while the other animals received distilled water, as suggested in a previous study [45]. Three days before sacrifice, the rats were fasted overnight, and an oral glucose tolerance test (OGTT) was conducted. At the end of the experiment, the animals were sacrificed, and blood and tissue samples were collected for further experiments.

#### 4.5.1. Biochemical Assays

To assess the biochemical parameters, quantitative total plasma glucose, total cholesterol and triglycerides were determined using a commercially available enzymatic colorimetric assay kit obtained from Erba Lachema (Brno, Czech Republic). Furthermore, plasma insulin concentrations were obtained using a sandwich ELISA kit from LINCO Research (Merck, Germany). Homeostasis assessment of insulin resistance (HOMA index) was subsequently calculated by the following formula (Equation (2)) [46]:

(2) $$\mathrm{HOMA}\;\mathrm{index}=\frac{\mathrm{fasting}\;\mathrm{plasma}\;\mathrm{insulin}\;\left(µU/\mathrm{mL}\right)\times \mathrm{fasting}\;\mathrm{plasma}\;\mathrm{glucose}\;\left(\mathrm{mmol}/L\right)}{22.5}$$

#### 4.5.2. Determination of Liver Histopathology Using Hematoxylin and Eosin (H&E)

To assess liver morphology, the tissues were excised and fixed in 10% buffered formalin solution for 24 h. The samples were infiltrated with xylene, then embedded in paraffin and cut into 5 µm thick sections. Subsequently, samples were stained with hematoxylin and eosin (H&E) to evaluate liver morphology. A microscopic examination was performed, and photographs were taken using a light microscope.

### 4.6. Determination of Genes Related Hepatic Gluconeogenesis and Antioxidant Using Quantitative Polymerase Chain Reaction (qPCR)

Total RNA was purified from freshly isolated rat liver tissues and HepG2 cells using TRIzol reagent (Thermo Fisher Scientific, Waltham, MA, USA), according to the manufacturer’s instructions. The first-strand cDNA was obtained using the iScript cDNA synthesis kit (Bio-Rad, Hercules, CA, USA), and qPCR was performed using SYBR real-time PCR master mix (Bioline, London, UK) on an ABI 7500 system (Life Technologies, Grand Island, NY, USA). Forward and reverse primers were either designed or used as previously published and purchased from Macrogen (Seoul, Korea). The primer sequences are shown in Supplementary Table S3 [47,48,49,50,51]. Gene expression was normalized to either actin for rat liver tissues or GAPDH for HepG2 cells. The mRNA level is reported as relative fold change (RFC), and qPCR amplification was performed in duplicate for each cDNA.

### 4.7. Determination of Proteins Related to Insulin Signaling Using Western Blot Analysis

HepG2 cells were lysed using CelLytic^TM^ MT mammalian tissue lysis/extraction reagent containing a 1% complete protease inhibitor mixture (Merck, Darmstadt, Germany) according to the manufacturer’s protocol. Samples were disrupted using a homogenizer and centrifuged at 5000× *g* for 10 min at 4 °C (total protein lysates). The protein concentration in each sample was determined using the Bradford assay (Bio-Rad, Hercules, CA, USA). Samples were stored at −80 °C prior to use.

For electrophoresis and Western blotting, total protein lysates were resolved in 2 × Lemmli solution and electrophoresed on a 10% sodium dodecyl sulfate polyacrylamide gel (SDS-PAGE), then transferred onto a polyvinylidene difluoride (PVDF) membrane (GE Healthcare, West Milwaukee, WI, USA). Nonspecific binding was then eliminated by blocking with 5% (*w*/*v*)non-fat dry milk in Tris-buffered saline containing 0.05% Tween-20 (TBS-T) for 1 h and subsequently incubated overnight with either polyclonal anti-rabbit or mouse antibodies (see detailed in Figure legends). The PVDF membrane was washed with TBS-T buffer and incubated with a horseradish peroxidase-conjugated ImmunoPure secondary goat anti-rabbit or anti-mouse IgG (Merck, Darmstadt, Germany) for 1 h. Proteins were detected using Super Signal West Pico chemiluminescent substrate (GE Healthcare, West Milwaukee, WI, USA) and quantitatively analyzed using the Image J program from the Research Services Branch (RSB) of the National Institutes of Mental Health (NIMH, Bethesda, MD, USA).

### 4.8. Statistical Analysis

Data are expressed as mean ± SEM. Statistical differences were assessed using one-way ANOVA followed by the LSD post hoc test or Dunnett’s test using the Statistical Package for the Social Sciences (SPSS) version 11.5 (SPSS Inc., Chicago, IL, USA). *p* Values < 0.05 were considered to be significant.

## 5. Conclusions

The current research indicates that TTE has antihyperglycemic, antihypertriglyceridemic, anti-insulin resistance, and hepatoprotective effects, mainly through suppressing hepatic gluconeogenesis. Antidiabetic effects of TTE in this study were clearly demonstrated by the reduction of PEPCK and G6Pase expression and activation of Akt/AMPK signaling pathway followed by inhibition of hepatic glucose overproduction in type 2 diabetes. These outstanding effects of TTE indicate that TTE is a potential alternative strategy for the treatment of T2DM and prevention of hepatic complications, including non-alcoholic fatty liver disease, non-alcoholic steatohepatitis, and cirrhosis that could be developed as pharmaceutical and/or nutraceutical products.

## Figures and Tables

**Table 1:** The composition of phenolic and flavonoid compounds in TTE analyzed by HPLC-PDA.

Active Compounds in TTE (Dry Weight) × (mg/g Extract)	
Gallic acid	0.053 ± 0.007
Catechin	ND
Epigallocatechin gallate	ND
Epicatechin	1.367 ± 0.025
Quercetin	0.134 ± 0.001

ND—non-detectable.

**Table 2:** Total phenolic contents and radical scavenging activities of TTE and its major components.

Active Compounds in TTE	Total Phenolic Content (mg GAE/g Extract)	ABTS Scavenging EC_50_ (µg/mL)	DPPH Scavenging EC_50_ (µg/mL)
Epicatechin	905.08 ± 12.92	22.49 ± 0.04	26.60 ± 0.34
Quercetin	938.25 ± 15.22	24.03 ± 0.34	15.04 ± 0.37
*Tiliacora triandra* (Colebr.) Diels leaf aqueous extract (TTE)	30.36 ± 0.16	324.20 ± 1.62	20.30 ± 0.05

**Table 3:** The effect of TTE on general characteristics and plasma parameters in type 2 diabetes mellitus (T2DM) rats.

	ND	ND + TTE	DM	DM + TTE	DM + Met	DM + TTE + Met
BW (g)	475.0 ± 7.2	452.0 ± 7.3	635.0 ± 7.3 *	510.0 ± 3.2 ^#^	525.0 ± 18.7 ^#^	480.0 ± 24.6 ^#^
Weight gain (g)	178.3 ± 10.1	171.3 ± 26.6	295.0 ± 6.7 *	185.0 ± 20 ^#^	210.0 ±20.0 ^#^	155.0 ± 45.0 ^#^
LW (g)	12.2 ± 0.1	11.3 ± 0.1	28.8 ± 7.3 *	16.6 ± 2.1 ^#^	19.6 ± 1.0 ^#^	17.1 ± 12.0 ^#^
Glucose (mg/dL)	116.6 ± 2.8	103.0 ± 2.1	408.2 ± 43.1 *	301.0 ± 24.1 ^#^	314.6 ± 34.5 ^#^	244.1 ± 11.6 ^##^
Insulin (ng/mL)	2.2 ± 0.8	2.1 ± 0.5	1.9 ± 0.7	1.8 ± 0.6	1.8 ± 0.9	1.7 ± 0.8
HOMA index	11.4 ± 0.8	9.7 ± 0.5	34.8 ± 1.8 *	24.6 ± 0.9 ^#^	24.3 ± 0.9 ^#^	18.9 ± 1.3 ^##^
Triglycerides (mg/dL)	67.0 ± 2.5	70.9 ± 3.7	104.0 ± 4.0 *	77.0 ± 0.9 ^#^	80.0 ± 2.1 ^#^	61.3 ± 0.9 ^##^

Data are presented as mean ± SE from 6 animals per group. Normal diet (ND), normal diet supplemented with TTE at a dose of 1000 mg/kg BW (ND + TTE), type 2 diabetes mellitus (DM), DM supplemented with TTE at a dose of 1000 mg/g BW (DM + TTE), DM treated with metformin at a dose of 30 mg/kg BW (DM + Met), and DM treated with TTE combined with metformin at doses of 1000 mg/kg BW and 30 mg/kg BW, respectively, (DM + TTE + Met). * *p* < 0.05 vs. the ND group, # *p* < 0.05 vs. the T2DM group. ## *p* < 0.05 vs. the DM + TTE group.

## Data Availability

The data presented in this study are available on request from the corresponding author.

## Supplementary Materials

The following are available online: Table S1 Retention time and parameters of calibration curve of standard phenolic and flavonoid compounds for HPLC-PDA method validation, Table S2 Gradient program for the HPLC analysis, Table S3 Primer sequences and expected amplicon sizes for gene amplification.
