# Supplementary material for: Tiliacora triandra (Colebr.) Diels Leaf Aqueous Extract Inhibits Hepatic Glucose Production in HepG2 Cells and Type 2 Diabetic Rats
Source: Molecules. 2021 Feb 25;26(5):1239. doi: 10.3390/molecules26051239 (PMC7956658; doi:10.3390/molecules26051239)
Supplement: Supplementary file 1 [file molecules-26-01239-s001.pdf]

molecules

MDPI

Article

Tiliacora triandra (Colebr.) Diels Leaf Aqueous Extract Inhibits Hepatic Glucose Production in HepG2 cells and Type 2 Diabetic Rats

Tipthida Pasachan [1], Acharaporn Duangjai [2], Atcharaporn Ontawong [2], Doungporn Amornlerdpison [3], Metee Jinakote [4], Manussabhorn Phatsara [5], Sunhapas Soodvilai [6,7] and Chutima Srimaroeng [1,*]

[1] Department of Physiology, Faculty of Medicine, Chiang Mai University, Chiang Mai 50200, Thailand; tp.pasachan@gmail.com
[2] Division of Physiology, School of Medical Sciences, University of Phayao, Phayao 56000, Thailand; achara.phso@gmail.com (A.D.); atcharaporn.on@up.ac.th (A.O.)
[3] Centre of Excellence in Agricultural Innovation for Graduate Entrepreneur and Faculty of Fisheries Technology and Aquatic Resources, Maejo University, Chiang Mai 50290, Thailand; doungpornfishtech@gmail.com
[4] School of Human Kinetics and Health, Faculty of Health Science Technology, HRH Princess Chulabhorn College of Medical Science, Chulabhorn Royal Academy, Bangkok 10210, Thailand; metee.jin@cra.ac.th
[5] Department of Anatomy, Faculty of Medicine, Chiang Mai University, Chiang Mai 50200, Thailand; msethadavit@gmail.com
[6] Research Centre of Transport Protein for Medical Innovation, Department of Physiology, Faculty of Science, Mahidol University, Bangkok 10400, Thailand; sunhapas.soo@mahidol.ac.th
[7] Excellent Centre for Drug Discovery, Mahidol University, Bangkok 10400, Thailand.
* Correspondence: chutima.srimaroeng@cmu.ac.th; Tel.: +66-53-935362; Fax.: +66-53-935365

Supplementary Table S1. Retention time and parameters of calibration curve of standard phenolic and flavonoid compounds for HPLC-PDA method validation.

Compound	Detected at Wavelength (nm)	RT (min)	Linear Range (μg/mL)	Regression Equation	Correlation Coefficient (r^2)
**Gallic acid**	280	6.45	0.5–100	y = 31428x − 15432	0.9999
**Catechin**	280	14.52	0.5–100	y = 7333.4x + 1054.7ǂ	0.9998
**ECGC**	280	17.63	0.5–100	y = 15109x − 14908	0.9993
**EC**	280	18.35	0.5–100	y = 7723.5x + 1562.9	0.9999
**Quercetin**	360	28.15	0.5–100	y = 22828x + 3052.2	0.9998

RT = retention time (min); y = peak area; x = concentration of standard (mg/l); r^2 = correlation coefficient of 3 data points in the calibration curve.

Supplementary Table S2. Gradient program for the HPLC analysis.

Times (min)	Solvents (%)		
	A (0.1 Phosphoric Acid)	B (Methanol)	C (Acetonitrile)
0	95	0	5
10	80	10	10
20	70	15	15
30	0	0	100
35	0	0	100

Supplementary Table S3. Primer sequences and expected amplicon sizes for gene amplification.

cDNA	Forward Primer (5′–3′)	Reverse Primer (5′–3′)	Amplicon Size (bp)
rCu-Zn SOD	GCAGAAGGCAAGCGGTGAAC	TAGCAGGACAGCAGATGAGT′	387
rGPx	CTCTCCGCGGTGGCACAGT	CCACCACCGGGTCGGACATAC	297
rCAT	CCTCCTCGTTCAAGATGTGGTTTTC	CGTGGGTGACCTCAAAGTATCCAAA	122
rPEPCK	CTCACCTCTGGCCAAGATTGGTA	GTTGCAGGCCCAGTTGTTGA	190
rG6Pase	AACGTCTGTCTGTCCCGGATCTAC	ACCTCTGGAGGCTGGCATTG	133
rActin	CCTAAGGCCAACCGTGAAAA	GGAGCGCGTAACCCTCATAG	181
h Cu-Zn SOD	AAAACACGGTGGGCCAAAG	GTGCGGCCAATGATGCA	141
hGPx	TTCCCGTGCAACCAGTTTG	TTCACCTCGCACTTCTCGAA	128
hCAT	TCATATACCTGTGAACTGTC	ATAGAATGACCGCACCTGAG	229
hPEPCK	AAGAGACACAGTGCCCATCC	ACGTAGGGTGAATCCGTCAG	201
hG6Pase	GAGACTGGCTCAACCTCGTC	CCTGGTCCAGTCTCACAGGT	139
hGAPDH	AGCCTTCTCCATGGTGGTGAAGAC	CGGAGTCAACGGATTTGGTCG	308

Cu-Zn SOD-copper zinc superoxide dismutase; GPx-glutathione peroxidase; CAT-catalase; PEPCK-Phosphoenolpyruvate carboxykinase; G6Pase- Glucose 6-phosphatase; GAPDH- Glyceraldehyde 3-phosphate dehydrogenase.
